# Practice towards Rational Drug Use at Finotselam and Asirade Zewudie Hospitals Based on WHO Core Drug Use Indicators, Northwest Ethiopia

**DOI:** 10.1155/2020/1634294

**Published:** 2020-08-28

**Authors:** Bekalu Dessie, Getachew Atalaye, Esubalew Diress, Alamirew Getahun

**Affiliations:** ^1^Department of Pharmacy, College of Health Sciences, Debre Markos University, Debre Markos, Ethiopia; ^2^Finoteselam Hospital, Finote Selam, Ethiopia

## Abstract

**Background:**

The rational use of drugs requires that patients receive medications appropriate to their clinical needs, in doses that meet their own individual requirements, for an adequate period of time, and at the lowest cost to them and their community with full information and with the lowest possible cost. If one of these is not met, it is referred to as irrational drug use. Many drugs have been sold or prescribed inappropriately in the world, and a significant part of the world population lacks access to essential medicine. The aim of this study was to assess practice towards rational drug use at Finoteselam and Asirade Zewudie hospitals.

**Methods:**

A cross-sectional study design was used for this study, and the study was conducted from October 11 to November 30, 2019. A total of 770 prescriptions were selected as per WHO criteria by using systematic random sampling and reviewed with the help of an observational checklist. The data were entered and processed with SPSS version 25 and evaluated using the WHO criteria.

**Result:**

The average number of drugs per prescription was 1.8 and 2.05, antibiotics encountered were 77.7% and 72.5%, injections encountered were 5.97% and 7.01%, percentage of drugs prescribed by generic names was 97% and 93.4%, counseling time was 1.6 minutes and 2.25 minutes, and dispensing time was 51.3 seconds and 62.72 seconds at Finoteselam and Asirade Zewudie hospitals, respectively.

**Conclusion:**

The majority of WHO core drug use indicators were not met in the two hospitals. The average number of drugs encountered in Asirade Zewudie hospital was slightly higher than the WHO recommended range, whereas the average number of drugs encountered in Finoteselam hospital was exactly equal. The percentage of antibiotics encountered was very high compared with WHO recommendation, but the percentage of injections encountered was below the WHO recommended range and time spent on counseling and dispensing was too short when compared with WHO recommendation. In addition to this, both hospitals had no essential drug list or formulary as well as a key drug list.

## 1. Introduction

Rational drug use is defined as giving an appropriate drug for the appropriate patient that must meet their clinical need, for an appropriate period of time, dose, and with the lowest possible cost [1–3].

World Health Organization (WHO) recommends three core drug use indicators. Prescribing indicators include the following parameters: average number of drugs prescribed, which is used to measure the number of drugs prescribed in a single prescription (degree of polypharmacy); percentage of drugs prescribed by generic names, which is used to measure the number of drugs prescribed by generic name; percentage of antibiotics encountered, which is used to assess antibiotic utilization; and percentage of injections encountered, which is important to measure prescriber attitude to injectable drugs. Patient care indicators address key aspects of what patients experience at a health facility and an evaluation method of interaction between patients and health workers. Patient care indicators include average consulting time, average dispensing time, percentage of drugs actually dispensed, percentage of drugs adequately labeled, and patient knowledge of correct dose [1].

Health facility indicators are important to measure the availability of formulary and key drug list in the facilities and include the availability of key drug and copy of essential drug list or formulary in the facility [4].

In the globe, 30–50% of drugs have been prescribed and sold inappropriately; moreover, one-third of the world population lacks access to essential medicine and the effect of antibiotic is compromised by the rapid escalation of antibiotic resistance combined with lack of novel antibiotics, and it is considered as global major health threats [5]. Global health expenditure was 6.5 trillion US dollars and 948 US dollars per person/year, and of this, 70–75% was spent on pharmaceutical procurement. In addition to consuming a majority of health expenditure costs, resistance to microbials is becoming prevalent in the world due to irrational use of medications [4, 6].

WHO estimates that more than half of the drugs are irrationally prescribed or dispensed and more than half of the patients fail to adhere to the prescribed regimen. Antibiotic resistance is a global problem which affects both developing and developed countries and causes hospitalization for a long period of time, and this results in more cost and this patient also at risk of acquiring a nosocomial infection, which is very difficult to treat and leads to increase treatment costs [7, 8].

Data on trends in medicine use showed that the average number of drugs used was increased from 1990 to 2003 from 2.2 to 2.7 per patient. Only 40–50% of patients were treated in compliance with the standard treatment guidelines. In developed countries, 4–10% of inpatients suffered from adverse drug reactions and this was the cause of 4–6% death in the USA and costs 130 billion US dollars and 466 million pounds sterling in the UK. According to WHO countries data 2002–2003, tuberculosis resistance, gonorrhea resistance to penicillin, pneumonia, and bacterial resistance to penicillin were 0–17%, 5–98%, and 0–70%, respectively [9].

A new resistance mechanism arises and spreads all over the world and threatens our ability to treat common infectious diseases. Without urgent action, we are heading to a postantibiotic era in which common infection and minor injury kill once again. It is accelerated by misuse and overuse of antibiotics. In each year in the USA, at least 2 million people are infected with antibiotic-resistant bacteria, and at least 23000 people die as a result. Today, drug resistance infection leads to approximately 700,000 deaths globally per year [4, 10, 11].

In Lesotho, polypharmacy was a problem in the outpatient department because the average number of drugs prescribed per encounter was 3.8, and of these, 37.6% were found to be antibiotic, adherence to STGs was 42.8%, and WHO recommends 100% generic prescreptions [8]. The drug utilization study in Pakistan was carried out, and the result was 24.63% which was not matched to diagnosis, dose of drug was inappropriate, and the duration of treatment was not specified [12].

Studies in Ethiopia showed that the majority of WHO standards were missed [1, 13–15]. The average consulting time was 5.1 ± 0.8 and 5.8 ± 1.06; dispensing time (minutes) was from 0.8 to 2.2 and 1.3 to 2.5 in primary health centers and health stations [16]. Study in southern Ethiopia showed that 2.33, 5.5, and 1.22 were drug per encounter, consulting time, and dispensing time, respectively, and the majority of patients (73.3%) knew the dose of prescribed medication [17].

In India, the practice of prescribing drugs by generic name was 42.9% and polypharmacy was a problem that is 52.7% of the analyzed prescription contained more than 3 drugs per prescription and 90% of the prescribed drugs were by their brand name [18–20]. In Yemen, prescribing by generic name, prescription encountered with antibiotics, and prescription encountered with injection were 39.2%, 66.2, and 46.2% [21]. In Jordan, the average number of drugs per prescription was 2.93. The percentage of encounters which had antibiotics or injections in the prescription was 17.7% and 8.1%, respectively [22].

As shown in the above literature studies, studies done in Ethiopia mainly focused on prescribing pattern. Thus, the aim of this study was to assess practice towards rational drug use at Finoteselam and Asirade Zewudie hospitals.

## 2. Method and Materials

### 2.1. Study Design

For this study, both prospective and retrospective cross-sectional study methods were used. In the study, all OPD prescriptions from October 11 to November 30, 2019, were included.

### 2.2. Sampling and Sampling Technique

Systematic random sampling was used, and 770 (385 from each hospital) prescriptions were included and 100 patients were prospectively selected and analyzed to assess patient care indicators as recommended by WHO [1].

### 2.3. Operational Definitions

- Antibiotics or antimicrobials are drugs that fight an infection caused by only bacteria
- Key drugs are selected items of drug or medicine which are preferable in the institution
- Adequate drug labeling means when the prescribed drug is expressed by generic name, strength, dose and frequency, quantity, expiry date, and date of dispensing if possible, and the batch number is also necessary
- Misuse and overuse of antibiotic: using antibiotic when it is not necessary and using more than prescribed or needed by diagnosis

## 3. Results

### 3.1. Prescribing Indicators

For this study, 770 (385 from each hospital) prescriptions were selected and analyzed. The average number of drugs encountered per prescription was 1.82 and 2.05 at FSH and AZH, respectively. Amoxicillin is the most commonly prescribed antibiotic in the two hospitals (Tables 1 and 2).

The percentage of prescriptions with antibiotics was 77.7% and 72.5% in FSH and AZH, respectively. The percentage of prescriptions with injections was 5.97% and 7.01% in FSH and AZH, respectively. From the total prescription surveyed, 19.2% and 28.3% contained three and more drugs.

**Figure 1:**
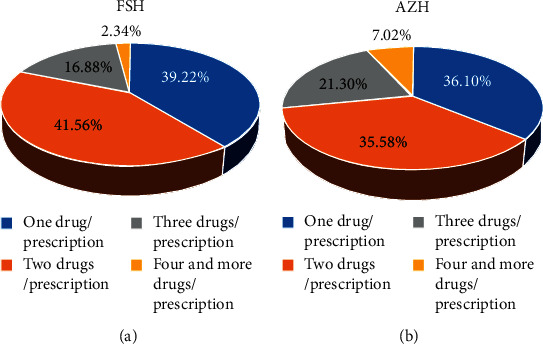
Number of drugs per prescription at (a) FSH and (b) AZH, from October 11 to November 30, 2019, northwest Ethiopia.

As presented in Figure 1, a single drug is prescribed in 151 and 139 prescriptions in FSH and AZH, respectively, 160 prescriptions contain 2 drugs in FSH, and 137 prescriptions contain two drugs in AZH.

97% and 93.4% of drugs were prescribed by generic names in FSH and AZH, respectively (Table 3).

### 3.2. Patient Care Indicators

244 and 260 antibiotics were prescribed, and among these, 92.2% and 94.2% were actually dispensed, and 95.6% and 96.25% were adequately labeled in FSH and AZH, respectively. The average dispensing time was 51.3 and 62.72 seconds at FSH and AZH, respectively. The average counseling time was 2.11 and 2.25 minutes at AZH and FSH, respectively (Table 4).

### 3.3. Facility Indicators

There was no EDL or any national drug formulary in both hospitals. In addition to this, these hospitals have not prepared key drug list which is very important to provide the minimum and common service for the community (Table 5).

## 4. Discussions

From this study, the average number of drugs encountered per prescription was 1.82 and 2.05 at FSH and AZH, respectively. The findings are lower when compared with 3.8 in Lesotho [8] and 2.7 in south India [19]. FSH result was found satisfactory with WHO recommendation, whereas the result from AZH is slightly higher than WHO recommendation which is 1.6–1.8 drugs encountered per prescription [2].

The percentage of prescriptions with antibiotics was 77.7% and 72.5% in FSH and AZH, respectively, which are higher compared with 37.6% in Lesotho [8], 9.6% in southern India [20], 57.8% in eastern Ethiopia [13], 66.2% in Yemen [21], 60.9% in Jordan [22], and also too much higher than the WHO recommended range which is 20–26.8% [2].

The percentage of prescriptions with injection was 5.97% and 7.01% in FSH and AZH, respectively, which is higher than 1.6% in southern India [19], lower than 10.6% in eastern Ethiopia [13], 46.2% in Yemen [21], and below the WHO recommended range which is 13.4–24.1% [2]. From the total prescription surveyed, 19.2% and 28.3% contained three and more drugs. These findings are lower than those reported in eastern Ethiopia (39.3%) [13] and Goa, India (52.7%) [20].

97% and 93.4% of drugs were prescribed by generic names in FSH and AZH, respectively. These findings are higher than those reported in southern India (42.9%) [19], southern Ethiopia (73.3%) [13], Goa, India (10%) [20], Yemen (39.2%) [21], southern India (42.9%), and eastern Ethiopia (90.6%).

244 and 260 antibiotics were prescribed, and among these, 92.2% and 94.2% were actually dispensed, and 95.6% and 96.25% were adequately labeled in FSH and AZH, respectively. This result is comparable with the finding in southern India where 96.6% and 99.3% were drugs actually dispensed and adequately labeled, respectively [19], and 75.77% and 3.3% were drug actually dispensed and actually labeled in eastern Ethiopia [13]. As the percentage indicated, AZH and FSH have better drug supply chain management system than the facilities described above. The average dispensing time was 51.3 and 62.72 seconds at FSH and AZH, respectively. This is lower than the dispensing time in southern Ethiopia (97.2 seconds) and in south India (138 seconds) but comparable with that in eastern Ethiopia (61.12 seconds). The average counseling time was 2.11 and 2.25 minutes at AZH and FSH, respectively, which is shorter compared with 3.7 minutes in southern India, 4.61 minutes in eastern Ethiopia, and 5.5 minutes in southern Ethiopia, and very short time when compared with 10 minutes of the WHO recommended standard [2]. There was no EDL or any national drug formulary in both hospitals. In addition to this, these hospitals have not prepared key drug list which is very important to provide the minimum and common service for the community.

## 5. Conclusion

In this study, majority of WHO core drug use indicators were not met in both hospitals. The average number of drugs encountered in AZH was slightly higher than the WHO recommended range, whereas that was exactly equal in FSH. The percentage of antibiotics encountered was very high compared with WHO recommendation, but the percentage of injection encountered was below the WHO recommended range and time spent for counseling and dispensing was too short when compared with WHO recommendation. In addition to this, both hospitals had no EDL or formulary as well as key drug list. Therefore, training, policy drafting, regulatory body revision, and establishing functional supply chain management should be done to overcome such irrational drug use and wasteful practice in the hospitals.

## Figures and Tables

**Table 1:** List of drugs prescribed frequently in AZH, northwest Ethiopia, from October 11 to November 30, 2019.

No.	Drug name	Dosage form	Strength	Frequency
1	Amoxicillin 500 mg	Capsule	500 mg	60 times
2	Ciprofloxacin 500 mg	Tablet	500 mg	47 times
3	Azithromycin 500 mg	Tablet	250 mg	33 times
4	Doxycycline 100 mg	Capsule	100 mg	32 times
5	Amoxicillin 125 mg/5 ml	Suspension	125 mg/5 ml	23 times
6	Cloxacillin 500 mg	Capsule	500 mg	20 times
7	Amoxicillin + clavulanic 125 mg + 31.5 mg/5 ml	Suspension	156.5 mg/5 ml	13 times
8	Cotrimoxazole 240 mg/5 ml	Suspension	240 mg/5 ml	10 times
9	Clarithromycin 500 mg	Tablet	500 mg	9 times
10	Amoxicillin + clavulanate (500 mg + 125 mg)	Tablet	500 mg + 125 mg	8 times

**Table 2:** List of drugs frequently prescribed in FSH, northwest Ethiopia, from October 11 to November 30, 2019.

No.	Drug name	Dosage form	Strength	Frequency
1	Amoxicillin 500 mg	Capsule	500 mg	68 times
2	Azithromycin 500 mg	Tablet	250 mg	39 times
3	Amoxicillin 500 mg + clavulanic 125 mg	Tablet	500 mg + 125 mg	30 times
4	Ciprofloxacin 500 mg	Tablet	500 mg	27 times
5	Amoxicillin 125 mg/5 ml	Suspension	125 mg/5 ml	18 times
6	Doxycycline 100 mg	Capsule	100 mg	17 times
7	Clarithromycin 500 mg	Tablet	500 mg	10 times
8	Cotrimoxazole 240 mg/5 ml	Suspension	240 mg/5 ml	9 times
9	Cloxacillin 500 mg	Capsule	500 mg	9 times
10	Amoxicillin (125 mg + clavulanic 31.5 mg)	Suspension	156.5 mg/5 ml	7 times

**Table 3:** Percentage of antibiotics and injections encountered from October 11 to November 30, 2019, at FSH and AZH, northwest Ethiopia.

No.	Prescribing indicators	FSH	AZH
1	Number of antibiotics prescribed	299	279
2	Number of injections prescribed	23	27
3	Total number of encounters	702	790
4	Percentage of antibiotics encountered	77.7%	72.5%
5	Percentage of injections encountered	5.97%	7.01%

**Table 4:** Patient care indicators from October 11 to November 30, 2019, at FSH and AZH, northwest Ethiopia (*n* = 100).

No	Patient care indicators	FSH	AZH
1	Average consulting time in minutes	2.11 minutes	2.25 minutes
2	Average dispensing time in seconds	51.3 seconds	62.72 seconds
3	Number of drugs actually dispensed	225	245
4	Total number of drugs prescribed	244	260
5	Percentage of drugs actually dispensed	92.2%	94.2%
6	Adequately labeled drug among actually dispensed	215	236
7	Percentage of drugs adequately labeled during dispensing	95.6%	96.3%

**Table 5:** Percentage of drugs prescribed by generic name from October 11 to November 30, 2019, at FSH and AZH, northwest Ethiopia.

No.	Prescribing indicator	FSH	AZH
1	Drugs prescribed by generic name	681	738
2	Total number of drugs prescribed	790	790
3	Percentage of drugs prescribed by generic name	97%	93.4%

## Data Availability

The data used to support the findings of this study are available from the corresponding author upon request (Bekalu D. Alamirew, bekiebda@gmail.com).

## Abbreviations

WHO: World Health Organization
CDC: Centers for Disease Control
RDU: Rational drug use
OPD: Outpatient department
STG: Standard treatment guideline
AZH: Asirade Zewudie hospital
FSH: Finoteselam hospital.
